# Preparation and Properties of Electrospun Phenylethynyl—Terminated Polyimide Nano-Fibrous Membranes with Potential Applications as Solvent-Free and High-Temperature Resistant Adhesives for Harsh Environments

**DOI:** 10.3390/nano11061525

**Published:** 2021-06-09

**Authors:** Hao-ran Qi, Deng-xiong Shen, Yan-jiang Jia, Yuan-cheng An, Hao Wu, Xin-ying Wei, Yan Zhang, Xin-xin Zhi, Jin-gang Liu

**Affiliations:** 1 School of Materials Science and Technology, China University of Geosciences, Beijing 100083, China; 2103200030@cugb.edu.cn (H.-r.Q.); 2003190024@cugb.edu.cn (Y.-j.J.); 2103190039@cugb.edu.cn (Y.-c.A.); 2003200021@cugb.edu.cn (H.W.); 2003200022@cugb.edu.cn (X.-y.W.); 3003200016@cugb.edu.cn (Y.Z.); 3003200015@cugb.edu.cn (X.-x.Z.); 2 Aerospace Research Institute of Materials& Processing Technology, Beijing 100076, China; shendx86@gmail.com

**Keywords:** polyimide, nano-fibrous membrane, electrospinning, adhesives, lap shear strength

## Abstract

High-temperature-resistant polymeric adhesives with high servicing temperatures and high adhesion strengths are highly desired in aerospace, aviation, microelectronic and other high-tech areas. The currently used high-temperature resistant polymeric adhesives, such as polyamic acid (PAA), are usually made from the high contents of solvents in the composition, which might cause adhesion failure due to the undesirable voids caused by the evaporation of the solvents. In the current work, electrospun preimidized polyimide (PI) nano-fibrous membranes (NFMs) were proposed to be used as solvent-free or solvent-less adhesives for stainless steel adhesion. In order to enhance the adhesion reliability of the PI NFMs, thermally crosslinkable phenylethynyl end-cappers were incorporated into the PIs derived from 3,3’,4,4’-oxydiphthalic anhydride (ODPA) and 3,3-bis[4-(4-aminophenoxy)phenyl]phthalide (BAPPT). The derived phenylethynyl-terminated PETI-10K and PETI-20K with the controlled molecular weights of 10,000 g mol^−1^ and 20,000 g mol^−1^, respectively, showed good solubility in polar aprotic solvents, such as *N*-methyl-2-pyrrolidinone (NMP) and *N,N*-dimethylacetamide (DMAc). The PI NFMs were successfully fabricated by electrospinning with the PETI/DMAc solutions. The ultrafine PETI NFMs showed the average fiber diameters (*d*_av_) of 627 nm for PETI-10K 695 nm for PETI-20K, respectively. The PETI NFMs showed good thermal resistance, which is reflected in the glass transition temperatures (*T*_g_s) above 270 °C. The PETI NFMs exhibited excellent thermoplasticity at elevated temperatures. The stainless steel adherends were successfully adhered using the PETI NFMs as the adhesives. The PI NFMs provided good adhesion to the stainless steels with the single lap shear strengths (LSS) higher than 20.0 MPa either at room temperature (25 °C) or at an elevated temperature (200 °C).

## 1. Introduction

**Figure 1:**
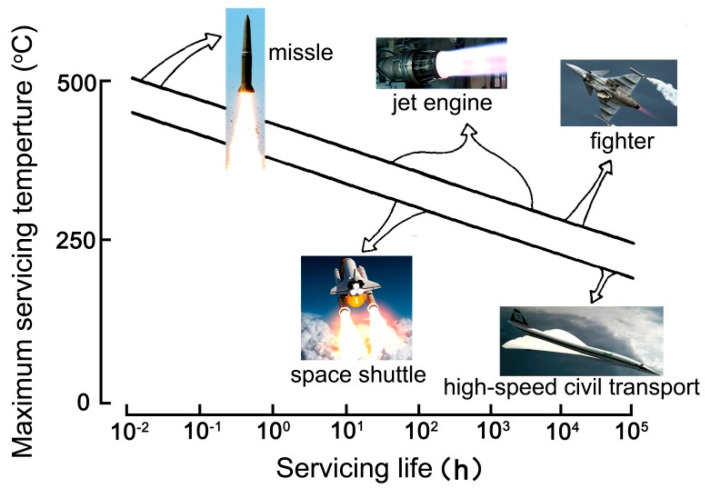
Potential applications of high-temperature adhesion in aerospace and aviation areas [4].

High-temperature bonding processes are widely used in modern industrial fields including aviation, aerospace, and microelectronics [1]. The use of high-temperature resistant adhesives is one of the most common procedures to achieve the high-temperature bonding procedure [2]. High-temperature-resistant adhesives generally refer to a class of special adhesives that can be used for a long time at hundreds of degrees centigrade or for a short time at temperatures over thousands of degrees centigrade [3]. They are usually used for structural bonding in supersonic missiles, launch vehicles, satellites, aircrafts and other components serving in harsh thermal environments, as shown in Figure 1 [4].

Among various high-temperature-resistant adhesives, including the inorganic non-metal ones and the organic high-performance polymers, the latter usually possess the better comprehensive properties, such as good mechanical properties, good processability, low cost, and so on [5]. For example, the heteroaromatic polymer adhesives, including polyimide (PI) and polybenzimidazole (PBI), can usually work continuously in a high-temperature environment above 250 °C and have been widely used in modern high-tech industry [6]. PI adhesives have been becoming one of the most promising materials for high-temperature adhesion in aerospace and aviation industries [7]. Up to now, the PI adhesives are mainly applied in the forms of liquid poly(amic acid) (PAA) precursors [8,9,10], preimidized PI varnishes, or solid B-staged PI films [11,12], or the thermoplastic PI powders [13]. The liquid-type PAA or PI adhesives are usually suffered from the low solid contents (usually less than 40 wt%) due to the limited solubility of the PAA or PI resins in the solvents, poor storage stability due to the degradation of PAA in thermal or humid environments, low adhesion efficiency due to the time-consuming curing procedures for PAA imidization, unsatisfied adhesion reliability due to the possible pinholes or voids existed in the PI adhesives caused by the releasing of solvent or water by-products in the PAA imidization, and so on. As for the powder-type PI adhesives, multiple-time adhesions are usually needed in order to achieve an acceptable adhesion layer thickness; meanwhile, the rheological parameters of the PI adhesives have to be carefully addressed so as to achieve good adhesion. Therefore, in practical applications, the current PI adhesive systems have to face various challenges both in formulations and in applying procedures. In the field of high-performance PI adhesives, the research and development of high-temperature-resistant PI adhesives without solvents or with few solvents has been become increasingly important [14].

In recent years, the rapid development of electrospun PI nano-fibrous membranes (NFMs) provides an instructive and valuable idea for developing solventless PI adhesives for high-temperature applications [15,16,17,18,19]. By analyzing the microscopic and macroscopic structural characteristics of PI NFMs, we can find that such materials possess high specific surface areas, high flexibility, highly entangled structure, and solvent-free characteristics, which are all required for high-performance PI adhesives [20,21,22,23]. Therefore, it can be anticipated that if the PI NFMs were used as adhesives, it is possible to achieve highly reliable adhesion for metal or other adherends. However, to the best of our knowledge, there are few reports in the literature of the application of PI NFMs in high-temperature adhesion fields.

In the current work, a series of electrospinning PI NFMs designed for high-temperature adhesion applications have been prepared and characterized as part of our ongoing efforts to develop high-performance PI NFMs for high-tech applications [24,25,26,27,28]. First, the flexible ether linkages were introduced into the molecular structure of the PIs via the ether-containing dianhydride and diamine monomers in order to afford good adhesion to metal substrates, which has been widely adopted for the development of PI adhesives. Secondly, thermally crosslinkable phenylethynyl (PE) end-cappers were incorporated into the molecular structure of the PIs in order to enhance the adhesion reliability of the PI NFMs, which was also widely conducted in the research and development of PI adhesives [29,30]. Thirdly, phenolphthalein groups were incorporated into the molecular structure of the PIs via the diamine monomer. Phenolphthalein groups possess a large molar volume, asymmetric molecular structure, polar carbonyl moiety, and the possibility of thermal crosslinking at elevated temperatures [31,32]. Thus, it could be expected that the PI NFM adhesives with the phenolphthalein groups might exhibit good processability, high heat resistance and high adhesion to metal substrates. As far as we know, the NFM-type PI adhesives used in high-temperature adhesion have been rarely reported in the literature. Therefore, the electrospun preparation and adhesion behaviors of the PE-terminated PI (PETI) NFMs with the phenolphthalein groups were performed in the current work. The soluble PI resin was prepared by introducing a phenolphthalein group into the PI molecular chain, and then the ultrafine fiber membrane was prepared by an electrospinning process with PI as the spinning material. The effects of the designed molecular structure on the thermal and optical properties of PETI NFMs were also studied.

## 2. Materials and Methods

### 2.1. Materials

4,4′-Oxydiphthalic dianhydride (ODPA, purity: 99.8%) and 4-phenylethynyl anhydride (PEPA, purity: 99.8%) were provided by Changzhou Sunlight Pharmaceutical Co. Ltd. (Changzhou, China) and dried in vacuo at 180 °C and 120 °C for 10 h, respectively prior to use. 3,3-Bis [4-(4-aminophenoxy)phenyl]phthalide (BAPPT) was synthesized in our laboratory according to the literature [33]. The high-purity solvents (purity > 99.5%), including *N,N*-dimethylacetamide (DMAc), *N,N*-dimethylforamide (DMF), *N*-methyl-2-pyrrolidinone (NMP), and dimethylsulfoxide (DMSO) were purchased from Sinopharm Chem Reagent Co. Ltd. (Shanghai, China) and distilled under reduced pressure with the dehydrating agents before use and stored under 4 Å molecular sieve.

### 2.2. Measurements

The inherent viscosity of the PETI resin dissolved in 0.5 g dL^−1^ NMP solution at 25 °C was measured by Ubbelohde viscometer (As-one, Osaka, Japan). The absolute viscosity of the PETI resin was also measured at 25 °C using Brookfield DV2-TRVCP viscometer (Middleboro, MA, USA) equipped with a CPA-41Z 115 conical spindle. The weight average molecular weight (*M*_w_) and number average molecular weight (*M*_n_) of PETI resins were measured by a gel permeation chromatography (GPC) system (Shimadzu, Kyoto, Japan). 1.0 g of the PETI resin was dissolved in 9.0 g of different test solvents (10% by weight solid content) and stirred for 24 h to test its solubility. Three solubility levels were defined by visual inspection: completely soluble (++), partially soluble (+−) and insoluble (−). The corresponding feature of completely soluble is a homogenous and clean state without phase separation, precipitation or gel formation. Insoluble is characterized by no change of the resin in appearance.

Attenuated total reflectance Fourier transform infrared (ATR-FTIR) spectra of PI membranes were recorded by an Iraffinity-1S FT-IR spectrometer (Shimadzu, Kyoto, Japan). Wide-angle X-ray (XRD) diffraction measurement is carried out by a Rigaku D/max-2500 X-ray diffractometer (Tokyo, Japan) under copper-Kα1 radiation, the working voltage was 40 kV and the working current was 200 mA. Field emission scanning electron microscopy (FE-SEM) imaging was observed using JSM-6700F (JEOL, Tokyo, Japan) under an acceleration voltage of 15 kV. Pt/Pd was spattered on the membranes before the measurement. The ultraviolet-visible (UV-Vis) spectra of PI fabric samples were measured using a Hitachi U-3210 spectrophotometer (Tokyo, Japan) at room temperature. The PI samples were dried at 100 °C for 10 h prior to the test. According to the procedure described in ASTM D1925 “Test method for yellowness index of plastics”, the yellow index (YI) values of the PI samples with the thickness of 50 μm were measured by using the X-rite color i7 spectrophotometer (Grand Rapids, MI, USA). The color parameters were recorded according to a CIE Lab equation. L^*^ is the lightness, where 100 means white and 0 means black. Positive a^*^ means red, and negative a^*^ means green. Positive b^*^ means yellow, and negative b^*^ means blue.

Thermogravimetric analysis (TGA) was measured by a TA-Q50 thermal analysis system (New Castle, DE, USA). The measurement was carried out in nitrogen at a heating rate of 20 °C min^−1^ in a temperature range of 50–760 °C. Differential scanning calorimetry (DSC) was performed in nitrogen at a heating rate of 10 °C min^−1^, the maximum temperature in the heating scanning was 400 °C, which was measured by TA-Q 100 thermal analysis system (New Castle, DE, USA). To evaluate the rheological properties, the PETI powder was pressed and molded to prepare the specimen discs (diameter: 25 mm, thickness: 1.5 ± 0.2 mm). The top parallel plate oscillated at a fixed angular frequency of 0.5 Hz and a fixed strain of 1.0%. The data collection range was 100~400 °C, and the heating rate was 4 °C min^−1^, which was measured by AR2000 rheometer (TA Instrument, New Castle, DE, USA). Single lap shear strength (LSS) was performed at a tensile speed of 2 mm min^−1^, which was measured by an Instron 5567 machine (Norwood, MA, USA).

### 2.3. Synthesis of PETI Resins

The PETI resins were synthesized from ODPA, BAPPT, and PEPA by a high-temperature polycondensation method. The detailed synthesis procedure could be presented by the synthesis of PETI-10K (designed molecular weight: 10,000 g mol^−1^). BAPPT (31.7013 g, 63.3 mmol) and NMP (190.0 g) were injected into a 500 mL three-necked flask equipped with a mechanical stirrer, an electric-heating bath, a Dean-Stark trap, and a nitrogen inlet at room temperature. Then, ODPA (18.0964 g, 58.3 mmol) was added to the solution. After stirring under the nitrogen flow for 5 h, PEPA (2.4823 g, 10.0 mmol) and additional NMP (19.0 g) were added into the solution together. The solid content of the reaction mixture was controlled to be 20 wt%. The reaction mixture was stirred for another 14 h at room temperature. Then, toluene (100 g) and isoquinoline (1.0 g) were added to the solution. The reaction mixture was heated to 180 °C for imidization reaction. During the reaction, the toluene azeotrope removed the water by-products at 140–145 °C for 16 h, and then toluene was distilled out of the reaction system until the internal temperature of the reaction reached 180 °C. The imidization reaction was carried out at 180 °C for 1 h and then cooled to 70 °C. The obtained viscous solution was precipitated into an excess of aqueous ethanol solution (10 wt%). The precipitated PETI-10K resin was dried at room temperature for 24 h, and was then further dried at 130 °C in vacuo for 24 h. The final PETI-10K resin was a pale-brown short filament. Yield: 47.62 g (95.2%).

The synthesis procedure of PETI-20K resin was similar to that mentioned above, except that the amounts of the reactants were 31.0997 g (62.1 mmol) for BAPPT, 16.1725 g (52.1 mmol) for ODPA, and 4.9646 g (20.0 mmol) for PEPA. Yield: 48.33 g (96.7%).

PI-ref (ODPA-BAPPT) resin without the phenylethynyl terminator was the synthesized procedure mentioned above, except the amounts of the reactants were 26.0781 g (52.1 mmol) for BAPPT and 16.1725 g (52.1 mmol) for ODPA. Yield: 39.41 g (97.3%).

### 2.4. Electrospinning Preparation of PETI NFMs

The detailed preparation procedure for the PETI NFMs could be illustrated by the electrospun preparation of PETI-10K NFM. The solution with a solid content of 46.5 wt% and an absolute viscosity of 8000 mPa·s was prepared by dissolving the well-dried PETI-10K resin in a DMAc solvent. Next, the obtained homogeneous PETI solution was loaded into a 5 mL syringe equipped with a needle-type nozzle with an inner diameter of 0.21 mm. A positive voltage of 16 kV and a negative voltage of −3 kV were applied between the injector and the collector, and the PETI-10K solution was extruded through the spinneret at a speed of 0.2 mL/h using a syringe pump. The distance between the spinneret and the grounded drum collector was maintained at 15 cm. The relative humidity in the electrospinning equipment was controlled at 50 ± 2%. The rotating speed of the collector was set to be 100 rpm. The PETI-10K NFM prepared by electrospinning was deposited on the aluminum foil as the support medium on the collector, and the obtained PETI-10K NFM was dried at 120 °C in vacuo for 1 h to remove the residual solvent.

The preparation procedure of PETI-20K NFM is similar to that of PETI-10K NFM. The solid content of the PETI-20K solution used for electrospinning is 37.0 wt% (absolute viscosity: ~8000 mPa·s).

### 2.5. Preparation of Stainless Steel Samples Adhered with the PETI NFMs

The size of the stainless steel specimens used for the LSS test is 100 mm (length) × 25.4 mm (width) × 2 mm (thickness). The specimens were pretreated with the standard sandpaper grinding procedure. The small pieces of PETI NFMs were stacked and placed between two stainless steel adherends. The size of these pieces was 12.5 mm (length) × 25.4 mm (width), and the thickness of the finally cured PETI NFMs was 0.3~0.5 mm. The lap area was 12.5 mm (length) × 25.4 mm (width). The pairs of stainless steel adherends containing the PETI NFMs were tightly fixed with clamps. Then, the specimens were placed in an oven and heated at 80 °C for 1 h, 150 °C for 1 h, 250 °C for 1 h, and 380 °C for 1 h to afford the bonded samples. The LSS values at room temperature were measured according to GB/T 7124-2008 (method of measuring tensile shear strength of adhesive (rigid material to rigid material)). The high temperature (250 °C) LSS test was carried out according to the GJB 444–1988 (adhesive high temperature tensile shear strength test method (metal to metal)). In each LSS test, six lap-shear specimens were tested at either room temperature (25 °C) or 200 °C, and the average data were recorded.

## 3. Results and Discussion

### 3.1. PI resins Synthesis and Electrospun PI NFMs Preparation

**Figure 2:**
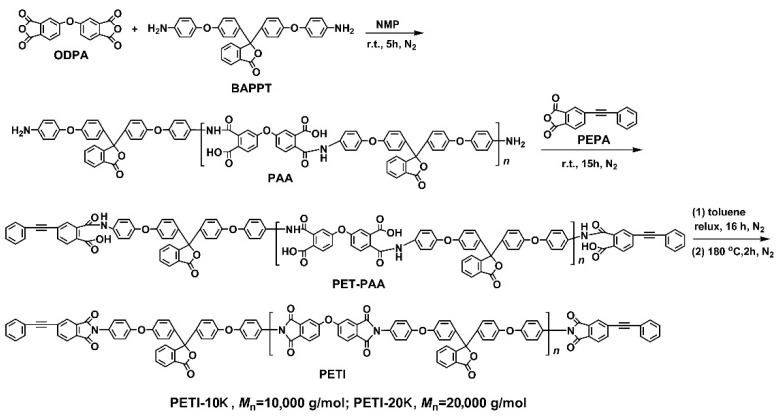
Synthesis of the phenylethynyl-terminated PI resins via a high-temperature polycondensation procedure.

Two phenylethynyl-terminated PI resins, PETI-10K and PETI-20K, with the controlled molecular weights of 10,000 g mol^−1^ and 20,000 g mol^−1^, respectively, were prepared according to the procedure shown in Figure 2. The phenylethynyl-terminated poly(amic acid)s (PET-PAAs) were first synthesized from ODPA, BAPPT and PEPA, which were then dehydrated at 180 °C to convert to the final PETI resins. For these two PETI resins, PETI-10K, with the relatively lower designed molecular weight, might possess a higher content of reactive phenylethynyl groups and lower melting viscosities than those of the PETI-20K analog. However, the mechanical properties of the cured PETI-10K might be lower than that of the PETI-20K counterpart with a higher designed molecular weight. Both of the PETI resins were soluble in the reaction medium and the polycondensation procedure was performed homogeneously. The continuous silky PETI-20K resin and the short rod-like PETI-10K resin were obtained, which indicates that the molecular weights of the resins were different.

The inherent viscosities ([*ƞ*]_inh_) and molecular weights of the PETI resins are listed in Table 1. The [*ƞ*]_inh_ values of PI resins were 0.43 dL g^−1^ for PETI-10K and 0.62 dL g^−1^ for PETI-20K, respectively. The moderate [*ƞ*]_inh_ values indicated the moderate molecular weights of the resins. The GPC measurement results shown in Table 1 could further verify the molecular weights of the resins. The PETI resins showed *M*_n_ values of 2.36 × 10^4^ g mol^−1^ for PETI-10K and 3.63 × 10^4^ g mol^−1^ for PETI-20K, respectively. These values were all higher than the designed molecular weights. This phenomenon was also reported in the literature [34]. This was thought to be due to the negative effects of the lithium bromide (LiBr) and phosphoric acid (H_3_PO_4_) in the GPC mobile phase to the solubility of the PETI resins in NMP. The PI-ref (ODPA-BAPPT) without a phenylethynyl end-capper showed a much higher [*ƞ*]_inh_ value (1.21 dL g^−1^) and molecular weights (*M*_n_: 19.0 × 10^4^ g mol^−1^; *M*_w_: 25.8 × 10^4^ g mol^−1^) than those of the PETI resins, indicating the excellent reactivity of the BAPPT diamine. Moreover, the PETI resins showed narrow PDI values of 1.47 for PETI-10K and 1.64 for PETI-20K, respectively.

**Figure 3:**
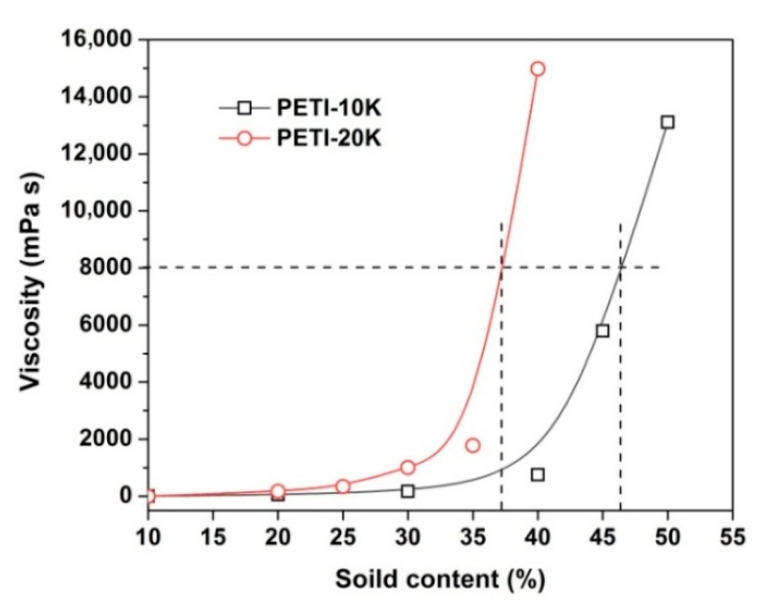
Viscosity-solid contents relationship of PI solutions in DMAc at room temperature.

As shown in Table 1, both of the PETI resins exhibited good solubility in polar aprotic solvents. These PETI resins were soluble in NMP, DMAc, DMF, and DMSO at room temperature with a solid content of 10 wt%. They were also partially soluble in chloroform and tetrahydrofuran (THF). For the PI-ref resin, it is completely insoluble in THF. Apparently, the good solubility of the PI resin in the test solvents is due to the controlled molecular weight, the flexible ether linkages and the bulky phenolphthalein group in the resin. Although both of the PI resins were all soluble in the polar aprotic solvents, the solubility of them in the specific solvent was different, which could be deduced from the viscosity-solid content relationship of PI solutions in DMAc, as shown in Figure 3. The PETI-20K solution showed a higher viscosity than that of the PETI-10K at the same solid content due to the higher molecular weight. This research is quite beneficial for determining the suitable solid contents of the PI resins for the following electrospinning fabrication. Generally, according to our previous research [20], a viscosity around 8000 mPa·s was suitable for the electrospun fabrication of PI NFMs via organo-soluble PI systems, by which ultrafine fibers without beads, spindle, and other drawbacks could usually be obtained.

**Figure 4:**
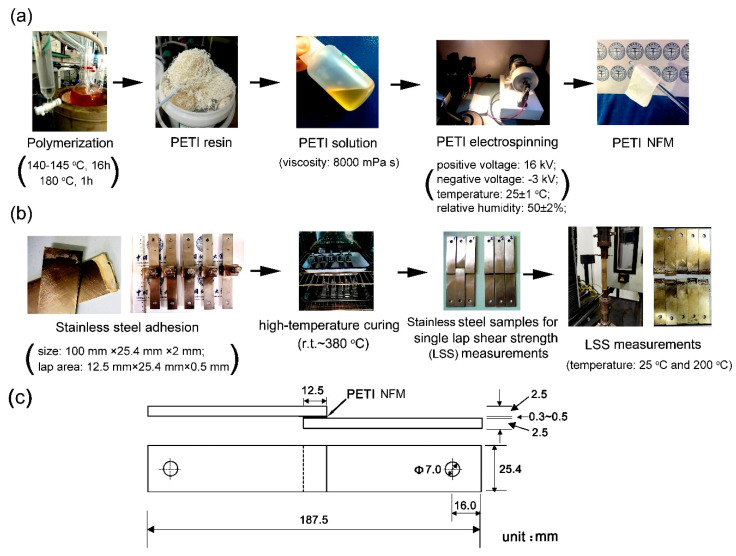
(**a**) Fabrication procedure of PI membranes via electrospinning; (**b**) Fabrication of stainless steel samples for lap shear strength (LSS) measurements; (**c**) Diagram for the sizes of adhesion samples.

**Figure 5:**
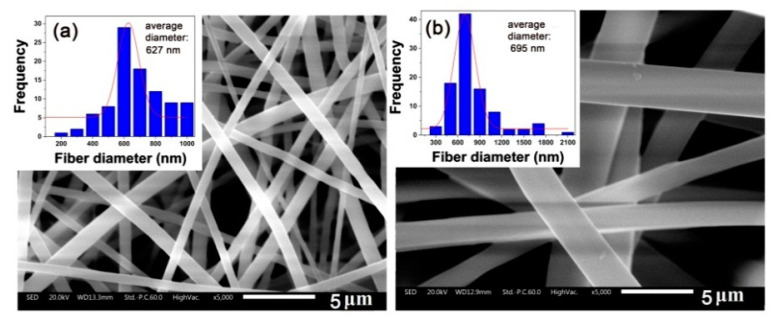
SEM images and average diameters (*d*_av_) of PI membranes. (**a**) PETI-10K; (**b**) PETI-20K.

According to Figure 3, in order to obtain the electrospinning solutions with a suitable viscosity (~8000 mPa·s), the solid contents of the PI solutions were about 46.5 wt% for PETI-10K and 37.0 wt% for PETI-20K, respectively. The PI NFMs were then fabricated by the electrospinning procedure shown in Figure 4a with the suitable conditions established in our previous work [20]. By adopting the suitable electrospinning conditions shown in Section 2.4, PI NFMs with fine micro-morphologies were successfully fabricated, as could be deduced from the SEM images of the PI NFMs shown in Figure 5. The obtained flexible and tough PI NFMs were composed of fine fibers. The average diameter of PETI-10K NFM was 627 nm, and the average diameter of PETI-20K NFM was 695 nm, respectively.

**Figure 6:**
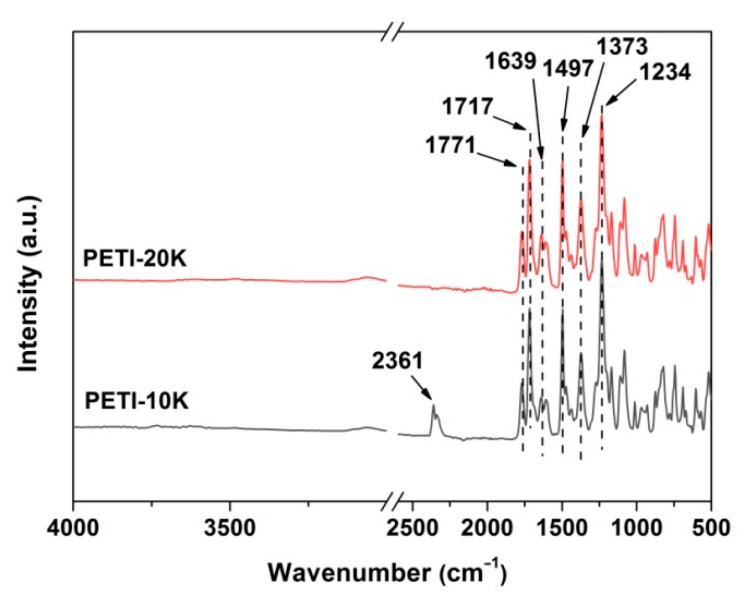
ATR-FTIR spectra of the electrospun PI nano-fibrous membranes.

AFR-FTIR measurements were carried out to confirm the chemical structures of the PI NFMs and the spectra of the PI NFMs were shown in Figure 6. According to the spectra, the characteristic absorptions of the imide rings were all clearly detected, in which 1771 cm^−1^ was the asymmetrical carbonyl stretching vibrations of the imide rings, 1717 cm^−1^ was the symmetric ones of the imide rings, and 1373 cm^−1^ was the C–N stretching vibration. In addition, for both PI NFMs, the characteristic absorptions of asymmetric stretching vibrations of –C(O)–O– groups in phthalide units at 1608 cm^−1^, the C=C vibrations in phenyl rings at 1502 cm^−1^, and the –O– vibrations at 1234 cm^−1^, were all detected in the spectra. The above information obtained by spectra confirmed the successful preparation of the PI NFMs.

### 3.2. Thermal Properties

**Figure 7:**
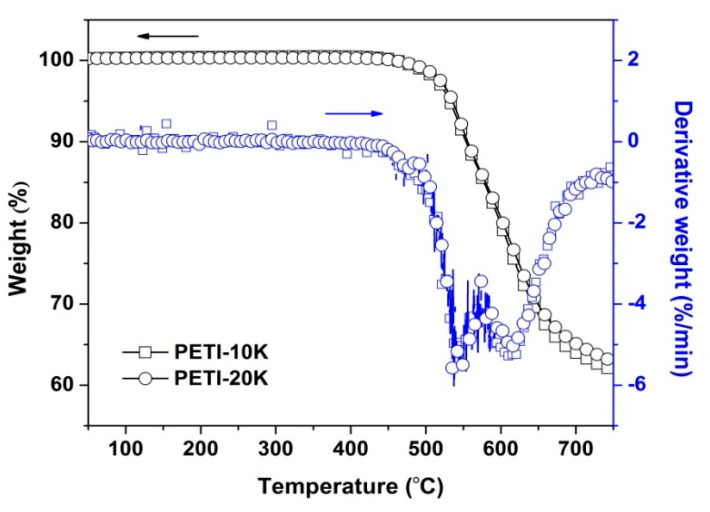
TGA and DTA plots of the electrospun PI nano-fibrous membranes in nitrogen.

The TGA and DSC measurements were performed to evaluate the thermal stabilities of the PI NFMs, and the results are tabulated in Table 2. The TGA and derivative TGA (DTG) plots of the PI NFMs were shown in Figure 7. Both of the PI NMFs showed good thermal stability up to 450 °C in nitrogen. With the further increase in the heating temperature, the PI NFMs began to decompose, and the 5% weight loss temperature (*T*_5%_) is 531.7 °C for PETI-10K NFM and 553.3 °C for PETI-20K NFM, respectively. Both PI NFMs exhibited two-staged thermal decomposition behaviors during thermal decomposition, which is reflected in the DTG plots. The first stage, which occurred around 540 °C, was thought to be the decomposition of the lateral phthalide units and the second ones around 600 °C could be attributed to the decomposition of the PI main chains. At last, the PI NFMs left about 64~65 wt% of their original weights at 700 °C (*R*_w700_). PETI-20K NFM with relatively higher molecular weights showed a slightly higher thermal stability than the PETI-10K NFM.

**Figure 8:**
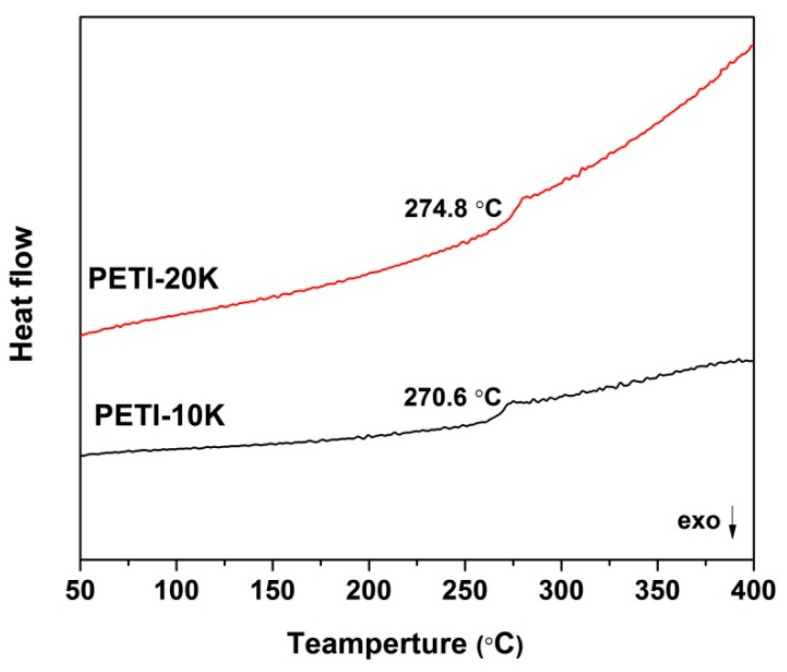
DSC curves of the electrospun PI nano-fibrous membranes in nitrogen.

Figure 8 depicts the glass transition behaviors of the PI NFMs recorded by DSC. All the PI NFMs exhibited good thermal stability, and the glass transition temperatures (*T*_g_s) were all higher than 270.0 °C. This was mainly due to the bulky phthalide units in the molecular chains of the polymers. The *T*_g_ values of PI NFMs were 270.6 °C for PETI-10K and 274.8 °C for PETI-20K, respectively. PETI-20K NFM with relatively higher molecular weights showed a slightly higher *T*_g_ value than PETI-10K NFM. The *T*_g_ values of the two PETI NFMs were inferior to those of the analogous PI-ref without molecular weight control. This indicated the critical influence of the molecular weights of the PIs on the movement of the molecular chain segments at elevated temperatures.

### 3.3. Adhesion Properties

**Figure 9:**
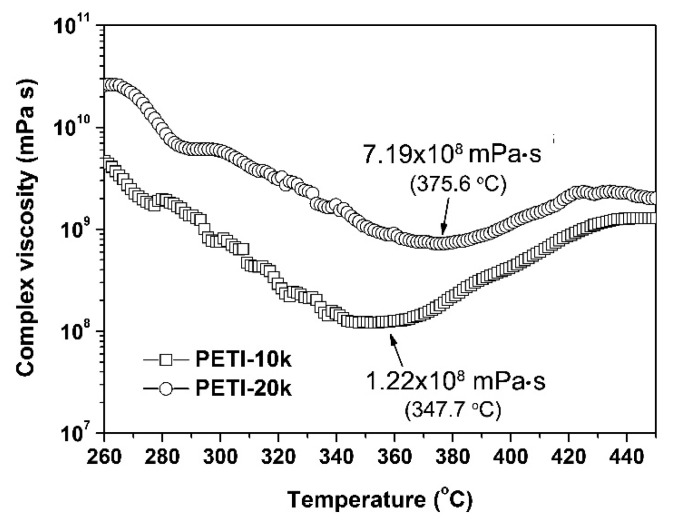
Rheological plots of phenylethynyl-terminated PI resins in nitrogen.

Single lap shear strength (LSS) tests were used to evaluate the adhesion properties of the current PETI NFMs to the stainless steel adherends. Before the LSS measurements, the rheological behaviors of the PETI NFMs were first investigated in order to provide essential information for the following adhesion evaluation. Proper melting flowability is usually required for the polymer adhesives so as to achieve good wetting between the adherends. Figure 9 records the rheological behaviors of the PI NFMs with the temperature range 260~450 °C, measured under the oscillation mode. Both of the PETIs showed good melting flowability, which was mainly attributed to the incorporation of flexible ether-linkage and the bulky phthalide units in the molecular chains. It could be clearly observed that the complex viscosity of the PETIs continuously decreased with the increasing temperature until a minimum viscosity (*η*_min_) value was achieved for 1.22 × 10^8^ mPa·s for PETI-10K NFM at 347.7 °C and 7.19 × 10^8^ mPa·s for PETI-20K NFM at 375.6 °C, respectively. PETI-10K NFM with relatively lower molecular weights showed a lower *η*_min_ value than PETI-20K NFM. Because of the cross-linking reaction of phenylethynyl groups, the complex viscosity of PETIs began to increase with the further increase of the test temperature. The information was quite beneficial for the following determination of the parameters for adhesion procedure.

**Figure 10:**
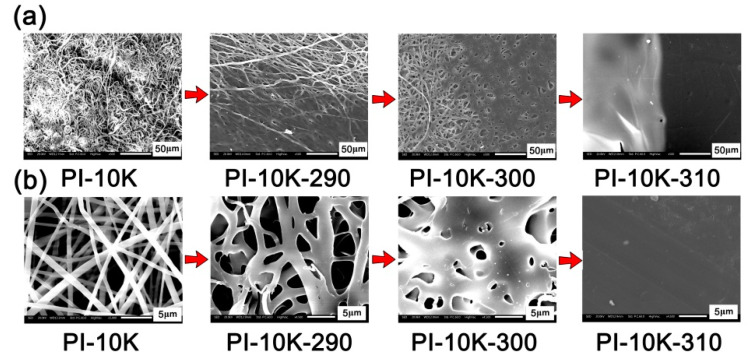
SEM images of electrospun PETI-10K nano-fibrous membranes at elevated temperatures. (**a**) ×500; (**b**) ×4500.

**Figure 11:**
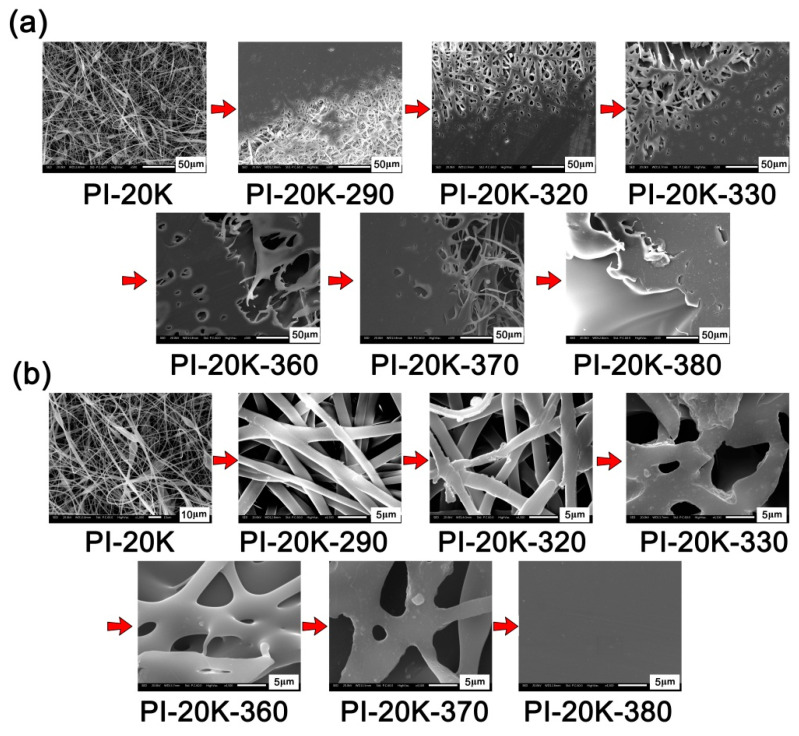
SEM images of electrospun PETI-20K nano-fibrous membranes at elevated temperatures. (**a**) ×500; (**b**) ×4500.

Secondly, the influence of the processing temperature on the melting flowability of the PI NFMs was further evaluated by the micro-morphologies investigation of the PI NFMs heated at elevated temperatures. Figure 10 and Figure 11 present the SEM images of PETI-10K and PETI-20K samples at elevated temperatures, respectively. It can be clearly seen that with the increase of the processing temperature, both PI NFMs began to fuse gradually. The difference was that the temperature of the complete fusion was different. For the PETI-10K sample, the UFM was almost completely molten when the temperature reached about 310 °C. However, for the PETI-20K sample, the temperature of complete fusion needed to reach 380 °C. This indicated that the PETI-10K NFM with a relatively lower molecular weight possessed a relatively lower processing temperature. This is consistent with the rheological test. However, it should be noted that the temperature of 310 °C was not high enough to induce the thermal crosslinking of phenylethynyl groups, which was unfavorable to improve the bonding strength of stainless steel adherends. Therefore, the final curing temperature of the two PETI NFMs was set to be 380 °C in the following adhesion research.

**Figure 12:**
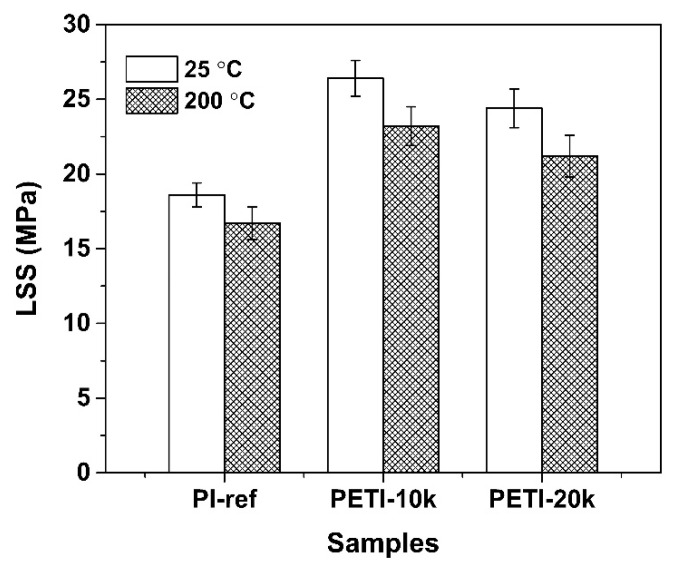
Lap shear strengths (LSS) of stainless steel adherends with electrospun PI nano-fibrous membrane adhesives.

At last, the PI NFMs were used as the adhesives to bond the stainless steel adherends according to the procedures shown in Figure 4b. The diagram for the sizes of the adhesion samples was shown in Figure 4c. The thickness of the PI NFMs was controlled to be 0.3~0.5 mm after high-temperature curing. The lap area was 12.5 mm (length) × 25.4 mm (width). The pairs of stainless steel adherends containing the PI NFMs were first tightly fixed with clamps and then the specimens were put into an oven. The adhesion was performed at elevated temperatures and at contact pressure. The thermal curing procedure (80 °C for 1 h, 150 °C for 1 h, 250 °C for 1 h, and 380 °C for 1 h) was determined according to the rheological feature of the PETIs. The LSS tests were carried out at room temperature (25 °C) and 200 °C, respectively. Figure 12 shows the LSS evaluation results of the stainless steel adherends with PI NFMs as the adhesives and the data are summarized in Table 2. Both room temperature LSS (LSS_25_) and high-temperature LSS (LSS_200_) values of the PETI NFMs were all higher than 20 MPa, which were higher than those of the PI-ref sample (LSS_25_ = 18.6 ± 0.8 MPa; LSS_200_ = 16.7 ± 1.1 MPa). It could also be seen from Figure 4b that the stainless steel adherends basically showed the cohesive failure mode instead of the adhesive failure mode. The PETI NMFs showed the average LSS_25_ values of 26.4 MPa for PETI-10K and 24.4 MPa for PETI-20K, respectively. The samples maintained about 87.9% and 86.9% of their original LSS values at 200 °C for PETI-10K and PETI-20K, respectively. On the other hand, the LSS values decreased with the order of PETI-10K > PETI-20K > PI-ref, indicating that PETI-10K NFM possessed the best adhesion to stainless steel adherends. From the results, one can deduce that the adhesion ability of the NFM-type PI adhesives might be affected by many factors, including the molecular weights, melting flowability, degree of crosslinking, and so on. The PETI-10K system possessed the best combination of properties, including a moderate *M*_n_ value, a low melting viscosity at the processing temperature, and crosslinking molecular chains, thus exhibiting the highest LSS values. On the other hand, few defects in the adhesion layer due to the solvent-free nature of the PETI NFMs were also critical for achieving a good adhesion ability. At last, the potential thermal crosslinking of the phthalide units in high-temperature air environments might also improve the adhesion ability of the PETI NFMs [31].

## 4. Conclusions

Two NFM-type phenylethynyl-terminated PI adhesives were developed and characterized in the current work. The derived PETI NFMs were endeavored to be used as the solvent-free adhesives for high-temperature adhesion applications. Experimental results indicated that the PETI samples showed a promising adhesion ability to stainless steel adherends. The LSS_25_ and LSS_200_ values of the samples were all higher than 20 MPa due to the solvent-free and thermally crosslinkable nature of the PETI NFMs. The PETI-10K sample possessed the best combination of properties, including good thermal stability (*T*_5%_ = 531.7 °C; *T*_g_ = 270.6 °C; *R*_w700_ = 64.0%), low melting viscosity (1.22 × 10^8^ mPa·s at 347.7 °C), and high adhesion strength for stainless steel adherends (LSS_25_ = 26.4 ± 1.2 MPa; LSS_200_ = 23.2 ± 1.3 MPa). The current research work might provide an instructive enlightenment for developing high-performance adhesives for high-tech applications. The detailed application results were being reported in our future work.

## Figures and Tables

**Table 1:** Inherent viscosities, molecular weights, and solubility of PI resins.

PI	[*ƞ*]_inh_ ^1^ (dL g^−1^)	Molecular Weight ^2^ (×10^4^ g mol^−1^)			Solubility ^3^					
		*M*_n_	*M*_w_	PDI	NMP	DMAc	DMF	DMSO	CHCl_3_	THF
PETI-10K	0.43	2.36	3.48	1.47	++	++	++	++	+−	+−
PETI-20K	0.62	3.63	5.95	1.64	++	++	++	++	+−	+−
PI-ref ^4^	1.21	19.0	25.8	1.36	++	++	++	++	+−	−

^1^ Inherent viscosities measured with PI resins at a concentration of 0.5 g dL^−1^ in NMP at 25 °C; ^2^ *M*_n_: number average molecular weight; *M*_w_: weight average molecular weight; PDI: polydispersity index (*M*_w_/*M*_n_); ^3^ ++: Soluble; +−: partially soluble; −: insoluble; CHCl_3_: chloroform; THF: tetrahydrofuran. ^4^ PI derived from ODPA and BAPPT.

**Table 2:** Thermal and adhesion properties of the electrospun PI nano-fibrous membranes.

Samples	*T*_5%_ ^1^ (°C)	*T*_10%_ ^1^ (°C)	*R*_w700_ ^1^ (%)	*T*_g_ ^1^ (°C)	LSS_25_^2^ (MPa)	LSS_200_^2^ (MPa)
PETI-10K	531.7	553.3	64.0	270.6	26.4 ± 1.2	23.2 ± 1.3
PETI-20K	535.3	555.2	65.1	274.8	24.4 ± 1.3	21.2 ± 1.4
PI-ref	514.1	527.6	59.0	288.7	18.6 ± 0.8	16.7 ± 1.1

^1^ *T*_5%_: 5% weight loss temperature; *T*_10%_: weight loss temperature; *R*_w7__00_: residual weight ratio at 700 °C in nitrogen; ^2^ LSS_25_: lap shear strength measures at 25 °C; LSS_200_: lap shear strength measures at 200 °C.

## Data Availability

Data is contained within the article.
